# Nothobranchius annual killifishes

**DOI:** 10.1186/s13227-020-00170-x

**Published:** 2020-12-15

**Authors:** Eva Terzibasi Tozzini, Alessandro Cellerino

**Affiliations:** 1 Stazione Zoologica “Anton Dohrn”, Napoli, Italy; 2 Scuola Normale Superiore, Pisa, Italy; 3 Leibniz Institute on Aging–Fritz Lipmann Institute, Jena, Germany

**Keywords:** Teleost, Life history adaptation, Aging, Diapause, Comparative genomics, Transgenesis, CRISPR/Cas9, Extreme habitat, RNA-seq, Neurodegeneration

## Abstract

Annual fishes of the genus Nothobranchius inhabit ephemeral habitats in Eastern and Southeastern Africa. Their life cycle is characterized by very rapid maturation, a posthatch lifespan of a few weeks to months and embryonic diapause to survive the dry season. The species *N. furzeri* holds the record of the fastest-maturing vertebrate and of the vertebrate with the shortest captive lifespan and is emerging as model organism in biomedical research, evolutionary biology, and developmental biology. Extensive characterization of age-related phenotypes in the laboratory and of ecology, distribution, and demography in the wild are available. Species/populations from habitats differing in precipitation intensity show parallel evolution of lifespan and age-related traits that conform to the classical theories on aging. Genome sequencing and the establishment of CRISPR/Cas9 techniques made this species particularly attractive to investigate the effects genetic and non-genetic intervention on lifespan and aging-related phenotypes. At the same time, annual fishes are a very interesting subject for comparative approaches, including genomics, transcriptomics, and proteomics. The *N. furzeri* community is highly diverse and rapidly expanding and organizes a biannual meeting.

## Natural habitat and life cycle

During the Monsoon season, many regions of the African Savannah are characterized by the formation of scattered ephemeral water pans of varying size, from a few square meters up to the extension of a small lake. These originate from the overflow of seasonal rivers and are characterized by progressively reduced size, rain-dependent duration, and strong excursions of water temperature and composition. Ephemeral ponds are inhabited by limited number of aquatic vertebrate species that coexist due to the evolution of specific, sometimes opposite, life strategies as adaptations to this erratic and inhospitable environment [1].

**Fig. 1:**
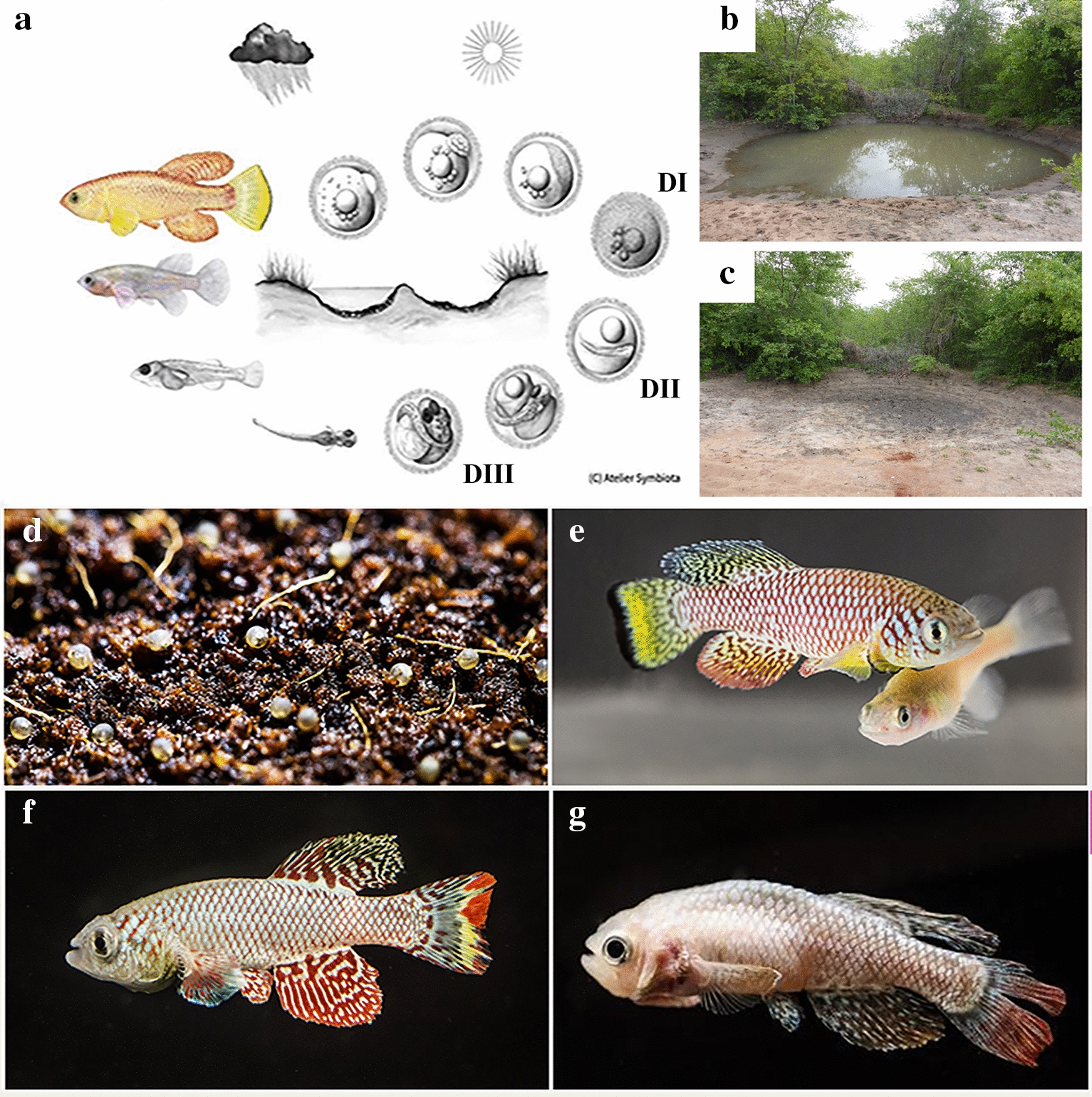
** a** Schematic drawing representing the typical life cycle of Nothobranchius fishes. Art by Atelier Symbiota, copyright Leibniz Institute of Aging, Fritz Lipmann Institute, with permission. **b** Example of an African pan in Mozambique during the Monsoon season, when Nothobranchius fishes are collected, and **c** the same African pan totally dried up, few month later, during the dry season (modified from [1] under CC 4.0)** d** Example of *Nothobranchius furzeri* eggs stored on peat moss. (Photo Nadine Grimm. Copyright Leibniz Institute of Aging, Fritz Lipmann Institute, with permission) **e** Young adult couple of *Nothobranchius furzeri*, sexually mature: the strong sexual dimorphism is apparent, with the intensely colored male and the dull female, in which a round abdomen indicates the presence of eggs. Photo Nadine Grimm. Copyright Leibniz Institute of Aging, Fritz Lipmann Institute, with permission. **f**,** g** Comparison between young and old phenotype: a young healthy, brightly colored male is shown in (**f**), in contrast to an old subject in (**g**), characterized by several features of the senescent phenotype, such as loss of colors, spinal curvature, body emaciation, and frayed fins. Photo Nadine Grimm. Copyright Leibniz Institute of Aging, Fritz Lipmann Institute, with permission

A notorious inhabitant of these ponds is the long-lived African lungfish (*Protopterus annectens*), which can survive the dry seasons thanks to its primitive lung and to its ability to enter in a state of quiescence (aestivation). This species is very long lived. Killifishes of the genus Nothobranchius evolved an opposite life history adaptation and are colloquially known as “annual fishes” in analogy to the life cycle of annual plants (Fig. 1a–c) [2]. Adult fish die at the end of the rainy season when the puddles desiccate. The embryos survive for months or years encased in the dry mud in a state of quiescence called "diapause" (Fig. 1d), a phenomenon also observed in the larvae of many insect species from temperate climates. When the monsoon rains replenish their habitat, eggs hatch and a new generation colonizes the ponds and breeds before the ponds disappear again [3].

Extensive field studies have been conducted in Southern Mozambique on the species *N. furzeri *[4] that shows the fastest maturation known in vertebrates (2 weeks) [5]. Adult females continue to produce eggs daily for all their life with no evidence of reproductive senescence in the wild [6]. The reproductive output is limited by habitat duration that in Southern Mozambique can last from a few weeks to a maximum of a few months [7]. The selective pressure exerted by the short duration of the habitat has pushed the evolution of a life history adaptation for fast growth and reproduction. This trait is coupled with an extremely short lifespan and rapid physiological decay (Fig. 1f, g) [3].

**Fig. 2:**
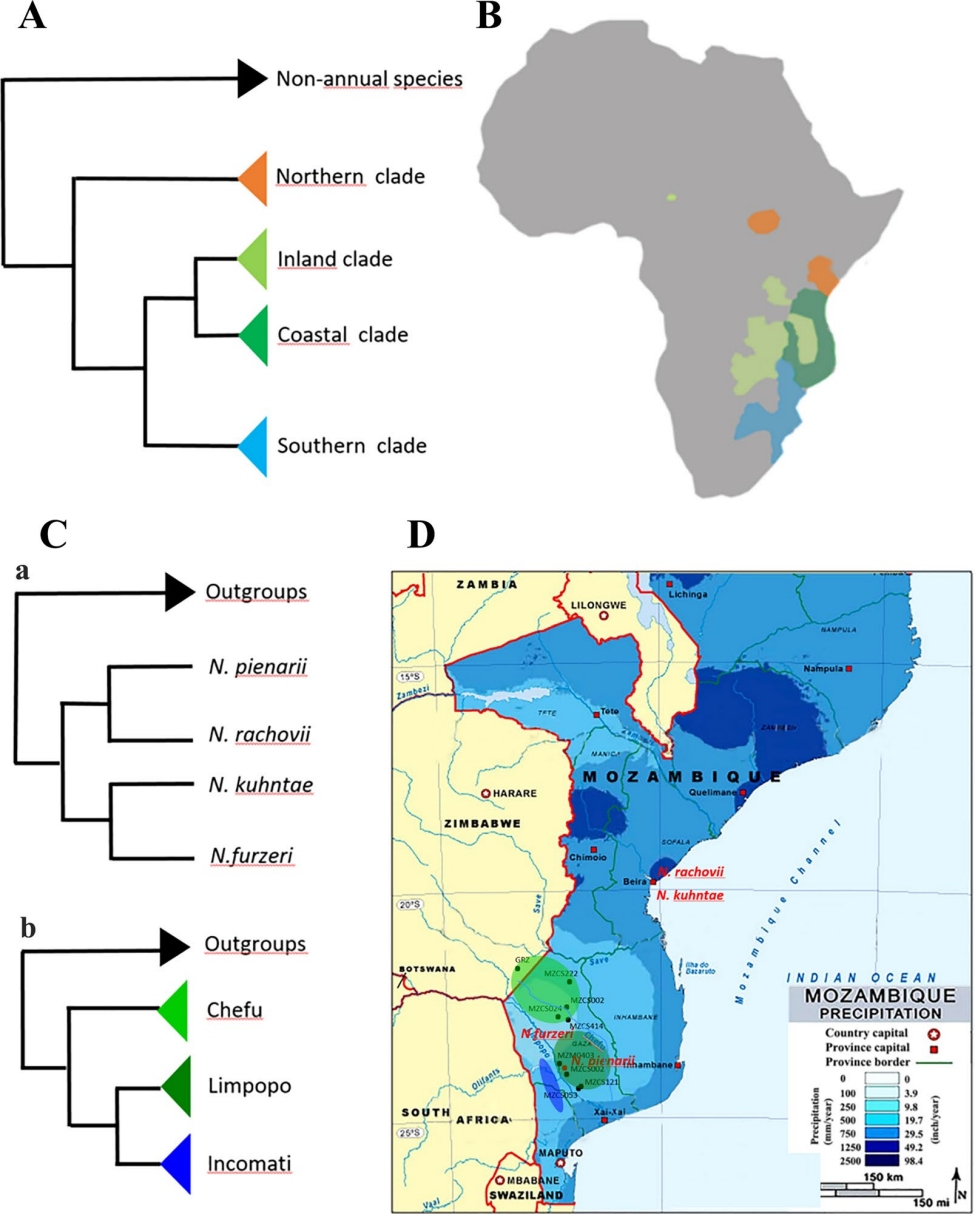
** a** Cladogram representing the relationship between the four principal clades of the Nothobranchius genus. Each clade is indicated with a different color. **b** Geographic map of Africa, the distribution of the four clades represented in the phylogenetic tree of panel A (modified from [9]) are indicated in the different colors. Note the almost complete lack of overlap between the ranges of the different clades. **c** Cladogram representing the relationship between the Nothobranchius species of the Southern clade whose lifespan characteristics are reported in Ref. [47] (top), and the relationship between the three geographic clades of *Nothobranchius furzeri* described in Ref. [8] (bottom). **d** Geographic map showing the distribution of the three Nothobranchius species described in the phylogenetic tree (**c** top), indicated as red points, and the distribution of the *Nothobranchius furzeri* populations described in (**c** bottom), indicated by black dots and specific sampling codes. The three colored ellipses identify the approximate range of the *N. furzeri* populations described in (**c**) overlaid onto the precipitation gradient (modified from [47])

Effective isolation of the pools has led to the formation of deeply genetically structured populations within the same species [8]. This structure offered the opportunity to study natural populations at the level of ecology, demography, and community, addressing the effects of aridity and population density on life history traits [1]. Indeed, the genus Nothobranchius includes almost 80 described species divided into four large clades [9] with a wide geographical distribution from KwaZulu-Natal to the White Nile in Sudan (Fig. 2a b). These clades are almost completely allopatric, indicating that geographic separation played the main role in speciation. The distribution range of Nothobranchius comprises inland arid regions and coastal areas characterized by much higher precipitations. In the Southern clade, different species are found in areas of different aridity (Fig. 2c, d). These morphologically defined species show deep genetic structuring with allopatrically separated populations [8, 10] spanning an aridity cline (Fig. 2c, d). This geographic distribution is of great relevance because aridity clines correlate with differences in captive lifespan [11, 12].

## Lab culture and field collection

Annual killifishes are of relative small size (most species ~ 5 cm) with clear sexual dimorphism (Fig. 1e). They can be bred in captivity by providing a suitable substrate (usually peat moss or coconut fibers) and incubating eggs and substrate outside of the water (Fig. 1d). They have no special requirement for water quality, but depend on a diet that is rich in protein and fat for reproduction, which can be provided by *Chironomus* spp. larvae [13]. The natural diet is varied and mainly consists of crustaceans [14]. Optimal temperature for breeding is 26 °C–28 °C. The main parameter that influences diapause is the temperature at which the eggs are incubated after collection [15]. Incubation above 28 °C inhibits diapause, whereas incubation at 24 °C causes a large fraction of embryos to enter diapause. Other factors, such as maternal age and substrate humidity, also influence diapause [16]. Fertilized eggs can be shipped in peat moss by normal post. There is a small, but well-connected, community of aquarium hobbyists dedicated to these colorful fishes that maintain and exchange large numbers of species. Laboratory stock of Nothobranchius were obtained via this channel already in the 1970s. The peculiar life cycle and extremely rapid aging of these animals, coupled with a relative ease of culture, has led them to become a new attractive experimental model, in particular, *N. furzeri* (Fig. 1e), which shows the shortest lifespan currently registered in captivity among vertebrates (3–7 months depending on genetic background) [4].

Field collection of adult animals is performed with seine nets shortly before the end of the rain season. Much of the field collection effort has concentrated in Southern Mozambique where up to three species can be found in sympatry and the aridity gradient is particularly steep (Fig. 2c).

## Major interests and research questions

### Aging phenotypes

The interest in Nothobranchius and particularly *N. furzeri* was sparked by the extreme short lifespan of this species, across the fields of aging, evolutionary, and developmental biology [3, 17–19].

**Fig. 3:**
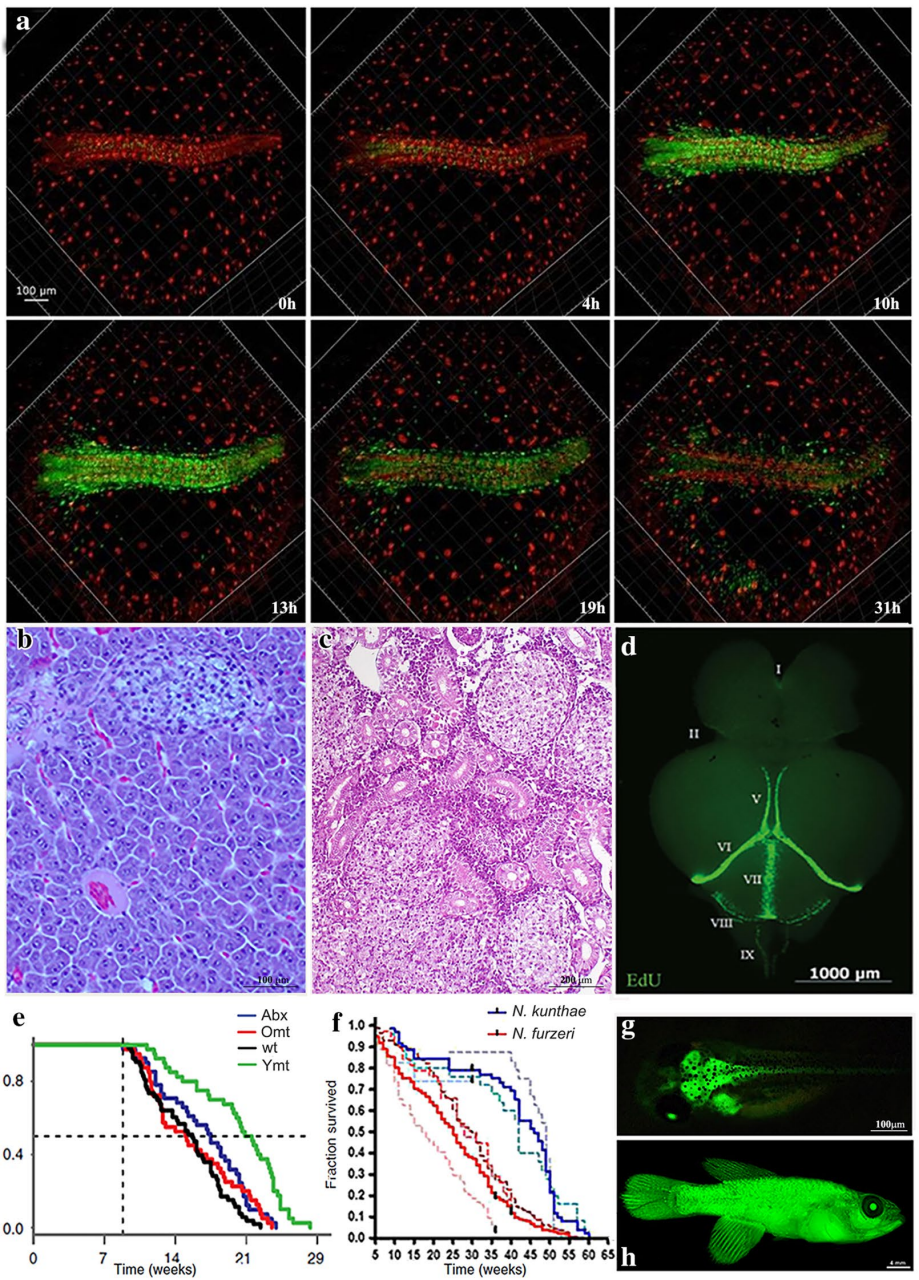
**a** Time lapse of release from diapause. Images are from a double-transgenic reporter line expressing the FUCCI system under the ubiquitin promoter: red and green fluorescent cells are in G1 and S/G2/M, respectively. Time is counted from the start of the video recording. Recording starts a 0 h when most cells are in G1. In the first few hours, there is low proliferative activity (4 h), followed by a proliferation burst between 15 and 18 h (10 h and 19 h), which gradually declines (31 h). From [53] reproduced under CC. Examples of spontaneous neoplastic lesions. **b** liver (20Xmagnification).** c** cephalic kidney (10Xmagnification). ** d** Whole-mount (WM) overview of Edu + cells in a 7-week-old *Nothobranchius furzeri* brain visualized 4 h after intraperitoneal injection to demonstrate the location of adult stem cell niches. Dorsal view of the entire brain. From [23] reproduced under CC. **e** Transferring young gut microbiome to adult fish prolongs life span. Black line control, red line homochronic (old to old) microbiome transfer late in life, purple line antibiotic treatment, and green line heterochronic (young to old) microbiome transfer late in life. Survival analysis. Statistical significance is calculated by Logrank test. * indicates a p value < 0.05; *** indicates a p value < 0.001. From [41] reproduced under CC—**f** Habit aridity influences evolution of lifespan in Nothobranchius**.** Survivorship of *N. furzeri* MZZW 07/01 strain (pink broken line *n* = 124), *N. furzeri* MZM 04/10 strain (red broken line, *n* = 113), *N. furzeri* MZCS 08/122 strain (brown broken line, *n* = 33), *N. kuhntae* MT-03/04 strain (light blue broken line *n* = 23; censored at age 33 weeks due to disease outbreak), *N. kuhntae* “aquarium strain” (blue broken line, *n* = 25), and *N. kuhntae* MOZ 04/07 strain (dark blue broken line, *n* = 24). Pooled survivorship of *N. furzeri* (*n* = 223), a species from arid habitats, is shown in solid red and the survivorship of pooled *N. kuhntae* (n = 72), the sister species from more humid habitats, is shown in solid blue. From [47] reproduced under CC. **g** Image of a transgenic tg(kif5aa:eGFP-sponge-29) f1 killifish embryo, 5 days after hatching. The transgene drives expression of GFP specifically in the brain. The green heart is driven by the cmlc2 promoter. Both promoters from zebrafish. From [42] reproduced under CC. **h** Image of GFP expression driven by zebrafish Ubiquitin promoter in the adult *N. furzeri*. From [53] reproduced under CC

In the biomedical field, *N. furzeri* is increasingly used as an experimental model. Studies have included investigations of a large number of age-related phenotypes at the integrative, cellular and molecular level. These include circadian rhythms [20], spontaneous tumorigenesis (Fig. 3b, c) [21], stem cell biology (Fig. 3d) [22, 23], regeneration [24], neurodegeneration [25], and heart and muscle biology [26, 27]. At the molecular level, studies have focused on mitochondrial function [28], epigenetics [26, 29], telomere attrition [30], lipid peroxidation [31], and expression changes at the genome-wide and proteome-wide levels [26, 29, 32–38]. These phenotypes are conserved to a significant degree between humans and *N. furzeri *[29, 39]*.* In addition, some aging phenotypes are more evident in *N. furzeri* than in murine models. As an example, *N. furzeri* shows a spontaneous age-dependent degeneration of some dopaminergic and noradrenergic neuronal populations in the brain associated with α-synuclein aggregation [25].

### Pharmacological interventions on aging

*N. furzeri* is attractive for biomedical researcher as a platform to investigate the effects of compounds or other interventions on lifespan and aging-associated phenotypes. The first molecule tested was the natural compound resveratrol, a natural polyphenol found in grapes and several species of berries, that in invertebrate models were shown to act as a sirtuin activator and induce life extension (Wood 2010). Resveratrol treatment results in prolonged lifespan and mitigated expression of aging markers in *N. furzeri*, probably due to its anti-inflammatory and anti-oxidant properties, but the molecular mechanisms remain unknown [40]. More recently, the life-extending effects of the complex I inhibitor rotenone was described [37]. Recently, *N. furzeri* was also used to investigate the effects of fecal microbiome transplantation on lifespan opening another area of interest (Fig. 3e) [41].

### Experimental genetics

Targeted genetic manipulations have allowed to investigate the effects of specific genes on lifespan and age-related pathologies. These techniques are particularly useful in combination with datasets of genome-wide expression profiling [11] whose analysis can deliver novel candidate genes/pathways. A first example was the identification and subsequent validation by transgenic techniques of the microRNA miR-29 as a novel regulator of age-dependent iron accumulation in the brain [42]. Several laboratories are currently investigating the age-related phenotypes of genetically modified *N. furzeri* lines. Specific knock-out lines that have been generated in this species are *Tert* −/− [43] and *Cbx7* −/− [44]. In the first case, the transgenic line was produced as a proof of concept of the CRISPR/Cas9-based genome editing approach; the gene was chosen to gain insights into the role of telomerase during aging (see also [44]). The second transgenic line was specifically created to investigate the role of Polycomb genes in diapause regulation [44].

### Evolutionary genetics

For evolutionary biologists, Nothobranchius fishes are of particular interest because evolution of aging in this taxon conforms to the expectations of the classical evolutionary theories on aging of Medawar [45] and Williams [46]. These theories posit that the strength of Darwinian selection decreases with age and becomes negligible at an age when the probability of survival and reproduction is small. This leads to the accumulation of mutations that reduce fitness late in life, as they are quasi-neutral. The rapidity by which the selection declines depends on mortality caused by external factors: if a species is subject to high predation, the window of selection is narrow because the probability of surviving declines rapidly. The theories therefore predict that an increase in extrinsic mortality results in the evolution of shorter lifespan. In killifishes, the window of selection is determined by the duration of the ponds. This effect of selection on aging is detected at three different levels: (i) annual lifestyle is a derived condition that evolved independently in several killifish lineages, while the basal condition is observed in non-annual killifishes that have a lifespan of several years [32], (ii) within the genus Nothobranchius, species originating from longer-lasting (more humid) habitats show longer captive lifespan and slower physiological decay than sister species from semiarid habitats (Figs. 2c, d, 3f) [47], and (iii) in multiple species from Southern Mozambique, population-scale differences in the progression of aging traits can be measured [11]. This allows to investigate genetic architecture of natural lifespan evolution by mapping of quantitative trait loci (QTLs) in populations with diverging phenotypes [48] and aging traits [48, 49]. This represented the main motivation for sequencing the *N. furzeri* genome [33, 34]. Analyzing the selection patterns identified convergent molecular evolution in short-lived annual killifishes and long-lived clownfishes [50, 51]. Very recently, it was also demonstrated that evolution of short lifespan in annual killifishes is connected to generalized and massive genome-wide relaxation of negative selection that affects 37% of the protein-coding genes and results in the accumulation of slightly deleterious mutations [32]. Remarkably, both relaxed selection and positive selection preferentially affected genes important for mitochondrial energy production [32]. This result can be interpreted as a relaxation of selection on traits associated with phenotypic maintenance and therefore longer lifespan, as predicted by Medawar’s theory [45].

### Developmental biology, diapause

Annual killifishes show three striking developmental traits [52]: (i) during embryonic cleavage, the cell cycle is extremely retarded as compared to non-annual sister taxa; (ii) gastrulation is characterized by dispersion and reaggregation of blastomeres; and (iii) development can be interrupted by diapauses at three specific developmental stages: the dispersed phase (DI), at mid somitogenesis (DII), and at the end of development before hatching (DIII) (Fig. 1a). Initial studies with reporter lines have visualized cell cycle dynamics during these phases, demonstrating that diapause exit is linked to a synchronized activation of the cell cycle (Fig. 3a) [53]. Very recently, a role for Polycomb in the regulation of diapause was revealed [44]. Several laboratories are currently investigating killifish embryology and the molecular control of diapause.

### Ecotoxicology

Assessing long-term consequences of toxicants on adult phenotypes and life history traits, such as fecundity in vertebrates, is a demanding task. *N. furzeri* has been used to test the effects of a number of pollutants [54, 55]

## Experimental approaches

### Compound testing

Nothobranchius fishes are convenient experimental models for life-long investigations on the effects of dietary and environmental manipulations. They are particularly suited to study the effects of compounds that can be administered via injections [38], with the food [40] or in the water [37] in the context of pharmacology or ecotoxicology. Similarly, long-term consequences of exposure to environmental toxins can be investigated. Recently, protocols for fecal microbiome transplantation were also established. In addition to lifespan, validated endpoints of analysis can be integrative phenotypes (locomotion, reproduction, and cognition), histopathological lesions, and several cellular/molecular phenotypes (e.g., lipofuscin).

### Genetic manipulations

Killifish eggs resemble medaka eggs, having a diameter of around 1 mm a yolk with lipid droplets and a very hard chorion covered by villi. They are amenable to microinjection and therefore genetic manipulations [43]. Overexpression of a specific construct can be obtained by random insertion of an expression cassette using Tol2 transposons [43]. The technique is quite efficient and 10–20% of correctly injected embryos can be expected to show mosaic expression. Promoter sequences isolated from the zebrafish genome such as bact (ubiquitous), cmlc2 (cardiomyocytes), ubi (ubiquitous), and kif5a (neurons) (Fig. 3g) drive a similar expression pattern in *N. furzeri* (Fig. 3g, h) [42, 53, 56]. They can be used to create reporter lines or to investigate the function of specific genes.

More recently, targeted gene knock-outs were obtained in *N. furzeri* by microinjection of Cas9 mRNA and the appropriate gRNAs, with efficiencies similar to those obtained in zebrafish [43]. Mutant lines can be generated by crossing F0 mosaic individuals with wild-type individuals and, using sequencing-based genotyping, mutants lines can be maintained in heterozygous condition.

### Quantitative genetics

The existence of laboratory strains differing in lifespan and other age-related phenotypes allows the genetic mapping of quantitative traits, by crosses between the strains and analysis of the F2 generation [33, 48, 49].

### Longitudinal studies

Nothobranchius fishes are also suitable for longitudinal studies where variations in molecular or physiological traits are correlated with individual lifespan [37].

## Research community and resources

### Nothobranchius symposium

A biannual “Nothobranchius symposium” is organized since 2014. In the last meeting, in 2018, around 100 delegates attended. The interests within the community are varied and include evolutionary biology, genetics/genomics, developmental biology, ecotoxicology, and biomedical research.

### Laboratory strains

The most widely used strain, named GRZ, is a highly inbred strain originating from a 1970 collection from the Gona Reh Zhou national Park in Zimbabwe. This strain has the shortest recorded lifespan.

Several wild-derived strains from different localities of Southern Mozambique differing in their age-associated phenotypes are also available. These originate mainly from a 2004 collection of A. Cellerino’s group [57], the collections of M. Reichard’s group [11], and D.R. Valenzano’s group [33]. There is lack of a general consensus on which of these longer-lived strains should become the standard and there is urgent need of a resource center that can maintain and distribute these strains, and in the future stable transgenic lines.

In addition, many different species/strains of Nothobranchius can be obtained via the national killifish associations, such as the American Killifish Association, the Deutsche Killifisch Gesellschaft, or the British Killifish Association.

### Genomic resources

Available resources include the genome of *N. furzeri* (https://nfingb.leibniz-fli.de/ and http://africanturquoisekillifishbrowser.org/) and several other killifish species, including non-annual species (BioProject ID: PRJNA531796), a BAC library (available from the Leibniz Institute on Aging, Fritz Lipmann Institute, Jena), extensive RNA-seq, and miRNA-seq databases for different organs (BioProject IDs: PRJNA412694, PRJNA553674, PRJNA503701, PRJNA379208, PRJNA358711), including longitudinal analysis (BioProject: PRJNA277768) and ChiP-seq of H3K4me3 (Small Reads Archive ID: SRP045718) and H3K27me3 (BioProject ID: PRJNA557479). Mass spectrometry-based proteomic data are under submission.

### Protocols and transgenesis

Detailed protocols were published for culture and generation of transgenic animals [13, 15, 43].

## Data Availability

Not applicable.
